# Genome-wide association mapping reveals novel sources of resistance to northern corn leaf blight in maize

**DOI:** 10.1186/s12870-015-0589-z

**Published:** 2015-08-20

**Authors:** Junqiang Ding, Farhan Ali, Gengshen Chen, Huihui Li, George Mahuku, Ning Yang, Luis Narro, Cosmos Magorokosho, Dan Makumbi, Jianbing Yan

**Affiliations:** National Key Laboratory of Crop Genetic Improvement, Huazhong Agricultural University, Wuhan, 430070 China; Institute of Crop Science, Chinese Academy of Agricultural Sciences, Beijing, 100081 China; Global Maize Program, International Maize and Wheat Improvement Center (CIMMYT), Apdo. Postal 6–641, 06600 Mexico, DF Mexico

## Abstract

**Background:**

Northern corn leaf blight (NCLB) caused by *Exserohilum turcicum* is a destructive disease in maize. Using host resistance to minimize the detrimental effects of NCLB on maize productivity is the most cost-effective and appealing disease management strategy. However, this requires the identification and use of stable resistance genes that are effective across different environments.

**Results:**

We evaluated a diverse maize population comprised of 999 inbred lines across different environments for resistance to NCLB. To identify genomic regions associated with NCLB resistance in maize, a genome-wide association analysis was conducted using 56,110 single-nucleotide polymorphism markers. Single-marker and haplotype-based associations, as well as Anderson-Darling tests, identified alleles significantly associated with NCLB resistance. The single-marker and haplotype-based association mappings identified twelve and ten loci (genes), respectively, that were significantly associated with resistance to NCLB. Additionally, by dividing the population into three subgroups and performing Anderson-Darling tests, eighty one genes were detected, and twelve of them were related to plant defense. Identical defense genes were identified using the three analyses.

**Conclusion:**

An association panel including 999 diverse lines was evaluated for resistance to NCLB in multiple environments, and a large number of resistant lines were identified and can be used as reliable resistance resource in maize breeding program. Genome-wide association study reveals that NCLB resistance is a complex trait which is under the control of many minor genes with relatively low effects. Pyramiding these genes in the same background is likely to result in stable resistance to NCLB.

**Electronic supplementary material:**

The online version of this article (doi:10.1186/s12870-015-0589-z) contains supplementary material, which is available to authorized users.

## Background

Maize (*Zea mays* L.) is an important crop for food, feed and industry. Moreover, it is a model genetic system with many advantages, including its great levels of phenotypic and genetic diversity [1]. Identifying the natural allelic variations that lead to this phenotypic diversity will contribute to the improvement of agronomic traits in maize breeding. However, dissecting quantitative traits poses numerous challenges that make gene identification more difficult, including the limitations of molecular biology and bioinformatics tools [2]. Rapid developments in genome-wide association mapping, combined with an extensive array of genome resources and technologies, have increased the power and accuracy to dissect complex traits and identify alleles associated with quantitative trait loci (QTL) for important agronomic traits [1, 3]. Recently, association mapping has become an influential approach for dissecting complex traits of interest. Distinct from the genetic analyses in segregating populations, genome-wide association study (GWAS) is based on the accurate phenotyping of a particular trait in a huge set of individuals that are widely unrelated (i.e., they have little or no family structure). For this reason, association mapping has been extensively used to study the genetic bases of complex traits in plant and animal systems [1, 4, 5].

Dissecting the genetic bases of different traits is the foundation of trait improvement; however, despite the recent advancements in this area, very little is known about the genetic architecture of many adaptive traits in maize [6], especially resistance to northern corn leaf blight (NCLB) and several other diseases. NCLB is caused by a hemibiotrophic fungal pathogen, *Exserohilum turcicum* (teleomorph *Setosphaeria turcica*) [7]. This disease is prevalent in maize growing areas worldwide and is associated with moderate-to-severe yield losses [8]. A severe NCLB infection prior to flowering may cause > 50 % losses in maize final yields [9]. The most economical and effective strategy for managing NCLB is the use of genetic resistance. The genetics of NCLB resistance have been extensively studied using biparental populations but are still poorly understood because of several factors, including low marker densities and the small population sizes used in many studies. A QTL analysis typically produces a large confidence interval, and it is usually uncertain whether a QTL corresponds to one or multiple linked genes [10, 11]. Until recently, only a small number of causal genes underlying large-effect QTLs have been identified and cloned in cereals [6].

In view of the potential power of association mapping to dissect the genetics of complex traits, and the problems of QTL mapping, this study was undertaken to shed light on the genetic architecture of NCLB resistance and to identify resistance-associated genes in globally collected diverse maize germplasm.

## Results

### Phenotypic diversity

**Fig. 1:**
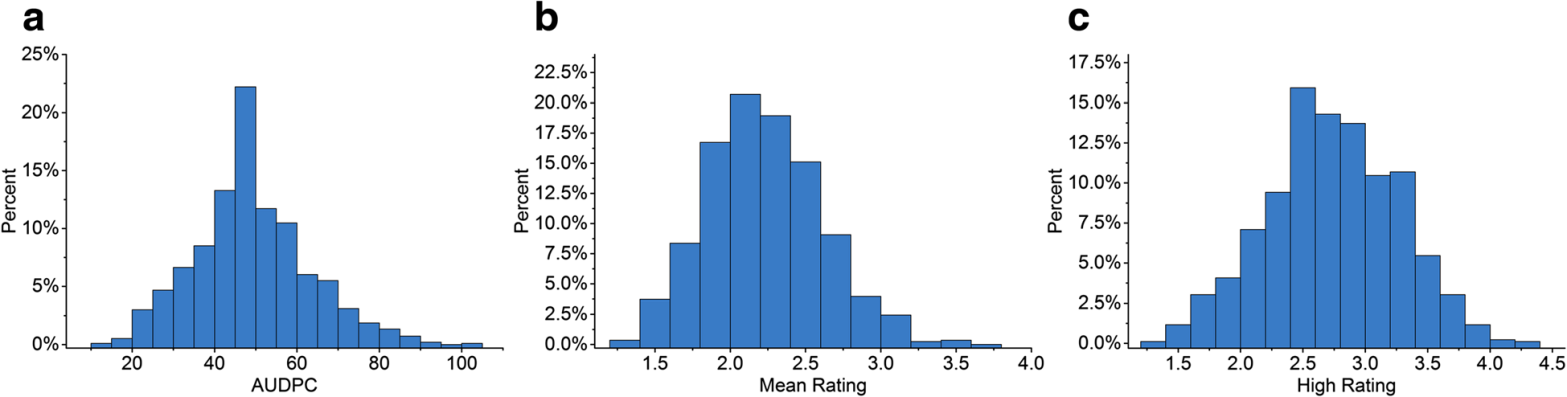
Frequency distribution of phenotypic variation of resistance to NCLB. The frequency distributions of area under disease progress curve (AUDPC), Mean Rating and High Rating are shown in **a**, **b** and, **c**, respectively

A global collection of 999 diverse inbred lines from the International Maize and Wheat Improvement Center (CIMMYT) germplasm collection was used for association mapping (Additional file 1: Table S1). Three related NCLB traits, mean rating, high rating and the area under the disease progress curve (AUDPC), were adopted to comprehensively evaluate the resistance to NCLB in association panel in 12 environments (Additional file 2: Table S2). The analysis of variance for NCLB resistance revealed significant differences (P ≤ 0.01) and high heritabilities for all of the traits under investigation (Table 1). Correlation results showed high positive associations between these traits. A maximum correlation value of 0.99 was observed between the mean rating and AUDPC, whereas the lowest value (r = 0.93) was observed between the high rating and AUDPC. No line was observed to be completely resistant to this disease, and most of the lines fell into the middle category (Fig. 1). The five highly resistant inbred lines were CIMBL225, CML305, CIMBL399, CML483 and CIMBL269, whereas the most susceptible lines were CML130, CML112 and CIMBL43 (Additional file 1: Table S1). These lines can be used as controls in future NCLB phenotyping studies and as parents to develop biparental populations for molecular breeding and marker-assisted selection.

**Table 1:** Analysis of variance, heritability and correlation

Traits	Mean squares		H^2b^	Correlation		
	E^a^	G^a^		High rating	Mean rating	AUDPC
High Rating	116.98**	2.35**	0.83	1		
Mean Rating	112.44**	1.10**	0.76	0.94**	1	
AUDPC	424481.57**	2012.67**	0.76	0.93**	0.99**	1

**Significant at *P* ≤ 0.01

^a^Mean square values split into environmental and genotypic mean square (E and G)

^b^Stands for broad-sense heritability

### Familial relatedness among lines

The 56,110 markers used in this study were used in different analyses, including principal component analyses (PCA), structure (Q) and kinship (K) analyses, to determine the relationships among the individuals in this association panel. The first 10 principal components in this association panel were shown to control 14.7 % of the cumulative variance, with each of them account for 0.7 %-6.0 % of the phenotypic variance (Additional file 3: Table S3). We also analyzed the data using STRUCTURE software to determine familial relatedness, and three subgroups were observed with >50 % possibility in each group (Additional file 4: Figure S1a). The K analysis also revealed that the 56,110 markers controlled 42.3 %, 47.4 % and 53.8 % of the total genetic variance for AUDPC, mean rating and high rating, respectively (Additional file 4: Figure S1 b, c and d).

### Genetic basis revealed by GWAS

**Fig. 2:**
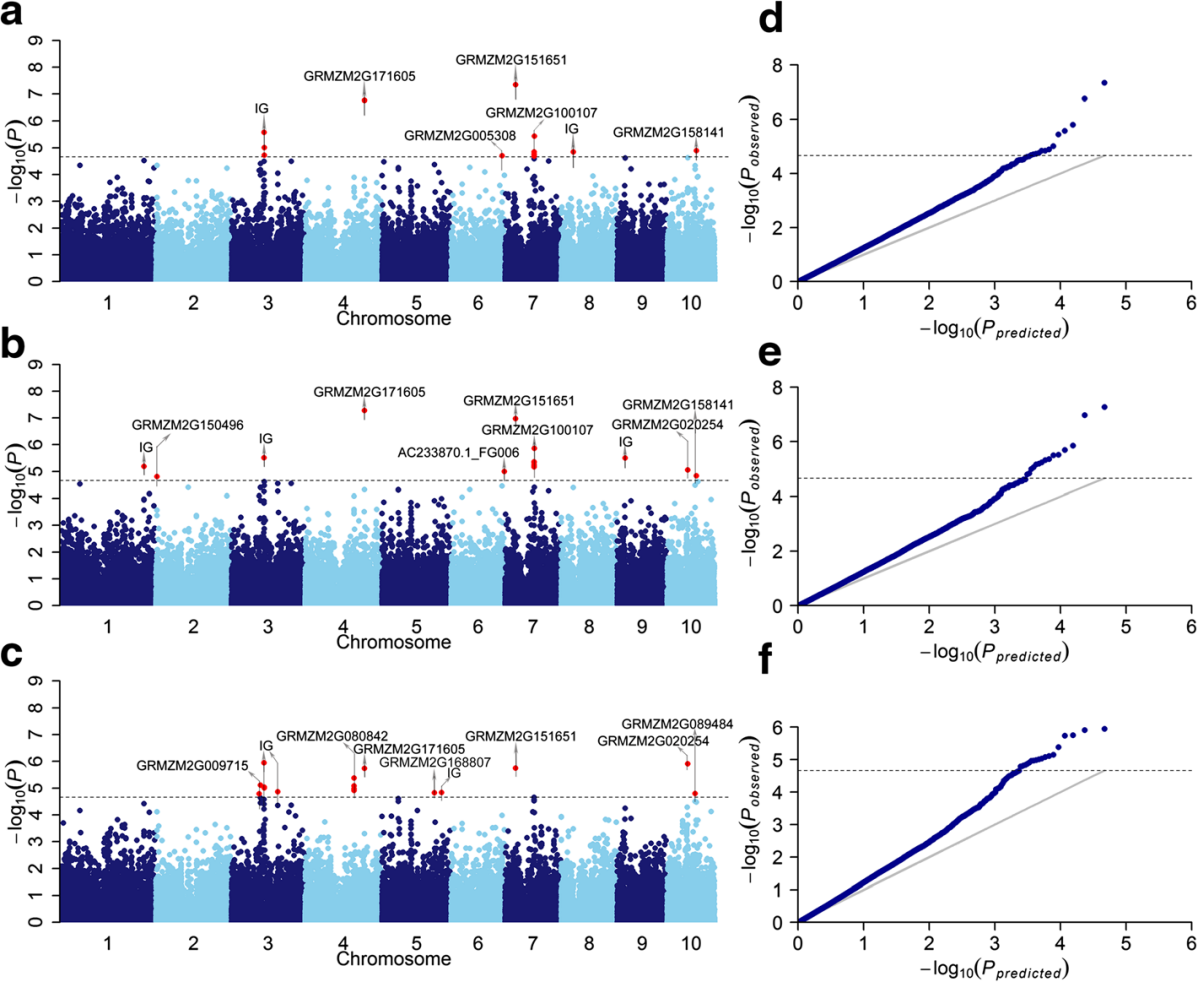
Manhattan plots and QQ plots resulting from the SNP-based GWAS for AUDPC, Mean Rating and High Rating. Manhattan plots for area under disease progress curve (AUDPC), Mean Rating and High Rating are shown in **a**, **b** and **c**, respectively. QQ plots for area under disease progress curve (AUDPC), Mean Rating and High Rating are shown in **d**, **e** and **f**, respectively. The genes that reach Bonferroni correction of P ≤ 2.15 × 10^−5^ are listed, and IG stands for intergenic which means no gene is identified

The SNP-based GWAS was performed using mixed linear model (MLM) with rare alleles (MAF < 5%) excluded, and both population structure (first 10 principle components) and kinship (K) were taken into account to avoid spurious associations. As is shown by the quantile-quantile plots (QQ plots) and Manhattan plots (Fig. 2), significant trait-marker associations that reached Bonferroni correction of P ≤ 2.15 × 10^−5^ (*P* < 1/n; *n* = total markers used) were observed. The number of significant markers revealed for AUDPC was 12, whereas 14 and 19 markers were associated with mean rating and high rating, respectively (Tables 2, 3 and 4). The number of significant loci varied from chromosome to chromosome, and each locus explained a small portion (2%-3%) of phenotypic variation. The maximum candidate loci were observed on chromosome 7 for the AUDPC and mean rating, whereas chromosome 3 and 4 each had seven significant loci for high rating. Based on the physical locations of significant SNPs on the B73 reference genome sequence, the concerning candidate genes lying in the significant loci were identified, which included five, seven and seven genes conferring resistance for AUDPC, mean rating and high rating, respectively. In total twelve unique genes were detected for at least one resistance trait. Five identical genes associated with two or three resistance traits were observed as revealed by their strong phenotypic correlations, which included one gene on chromosome 4 (GRMZM2G171605), two genes on chromosome 7 (GRMZM2G100107 and GRMZM2G151651) and two genes on chromosome 10 (GRMZM2G158141 and GRMZM2G020254). More importantly, functional annotations of the five genes showed that three of them related to plant defense. For example, GRMZM2G100107 was annotated as the SANT domain-associated protein, which played an important role in disease resistance [12, 13]. GRMZM2G158141 encoded antifreeze protein and may play direct role in plant defense [14]. GRMZM2G020254 encoded DNA-binding WRKY, which can *cis* regulate defense genes by signal transduction under biotic stress conditions [15].

**Table 2:** Candidate genes, chromosomal position and SNPs significantly associated with Area under Disease Progress Curve (AUDPC) detected by SNP-based GWAS

No.	Candidate gene	Chromosome	Physical position (AGP v.2)	SNP	Allele	P value	FDR*	MAF^a^	R^2^	Annotation
1	Intergenic	3	103166745	PZE-103062307	A,G	2.67E-06	0.031	0.09	0.02	
2	Intergenic	3	103544700	PZE-103062210	A,G	1.88E-05	0.074	0.17	0.02	
3	Intergenic	3	103769943	PZE-103062159	A,C	9.87E-06	0.074	0.13	0.02	
4	GRMZM2G171605	4	186590896	PZE-104110312	A,G	1.74E-07	0.004	0.18	0.03	4'phosphopante theinyl transferase
5	GRMZM2G005308	6	160053330	PZE-106113397	A,G	1.96E-05	0.074	0.24	0.02	U3 small nucleolar ribonucleoprotein
6	GRMZM2G151651	7	33447828	SYNGENTA5726	G,A	4.58E-08	0.002	0.08	0.03	
7	GRMZM2G100107	7	91683817	SYN16533	G,A	2.07E-05	0.074	0.44	0.02	SANT associated
8	GRMZM2G100107	7	91684720	PZE-107044973	A,G	1.74E-05	0.074	0.43	0.02	SANT associated
9	Intergenic	7	91686972	PZE-107044977	C,A	1.43E-05	0.074	0.44	0.02	
10	Intergenic	7	92335869	PZE-107045210	G,A	3.64E-06	0.034	0.21	0.02	
11	Intergenic	8	37657703	PZE-108032335	G,A	1.43E-05	0.074	0.36	0.02	
12	GRMZM2G158141	10	91956279	PZE-110049068	G,A	1.28E-05	0.074	0.09	0.02	Antifreeze protein

*False discovery rate-corrected p-values

^a^Minor allele frequency

**Table 3:** Candidate genes, chromosomal position and SNPs significantly associated with mean rating detected by SNP-based GWAS

No.	Candidate gene	Chromosome	Physical position (AGP v.2)	SNP	Allele	P value	FDR*	MAF^a^	R^2^	Annotation
1	Intergenic	1	264172677	PZE-101213762	C,A	6.44E-06	0.029	0.18	0.02	
2	GRMZM2G150496	2	3735379	PZE-102007366	G,A	1.56E-05	0.048	0.18	0.02	Inositol-pentakis-phosphate 2-kinase
3	Intergenic	3	103166745	PZE-103062307	A,G	3.05E-06	0.025	0.09	0.03	
4	GRMZM2G171605	4	186590896	PZE-104110312	A,G	5.33E-08	0.002	0.18	0.03	4'phosphopantetheinyl transferase
5	AC233870.1_FG006	6	167018912	PHM5529.7	C,A	1.01E-05	0.036	0.07	0.02	
6	GRMZM2G151651	7	33447828	SYNGENTA5726	G,A	1.06E-07	0.002	0.08	0.03	
7	GRMZM2G100107	7	91683817	SYN16533	G,A	5.79E-06	0.029	0.44	0.02	SANT associated
8	GRMZM2G100107	7	91684720	PZE-107044973	G,A	4.71E-06	0.027	0.43	0.02	SANT associated
9	GRMZM2G100107	7	91685110	SYN16536	G,A	6.81E-06	0.029	0.43	0.02	SANT associated
10	Intergenic	7	91686972	PZE-107044977	A,C	4.34E-06	0.027	0.44	0.03	
11	Intergenic	7	92335869	PZE-107045210	A,G	1.38E-06	0.021	0.21	0.03	
12	Intergenic	9	25257190	SYN28207	A,G	3.18E-06	0.025	0.1	0.03	
13	GRMZM2G020254	10	65416520	PZE-110034333	A,G	8.72E-06	0.034	0.21	0.02	DNA-binding WRKY
14	GRMZM2G158141	10	91956279	PZE-110049068	G,A	1.45E-05	0.048	0.1	0.02	Antifreeze protein

*False discovery rate-corrected p-values

^a^Minor allele frequency

**Table 4:** Candidate genes, chromosomal position and SNP significantly associated with high rating detected by SNP-based GWAS

No.	Candidate gene	Chromosome	Physical position (AGP v.2)	SNP	Allele	P value	FDR*	MAF^a^	R^2^	Annotation
1	GRMZM2G009715	3	87786034	SYN15223	G,A	1.63E-05	0.040	0.14	0.02	Potassium uptake protein TrkA
2	Intergenic	3	91910150	PZE-103066271	A,G	2.12E-05	0.051	0.05	0.02	
3	Intergenic	3	92149095	PZE-103066064	C,A	7.73E-06	0.039	0.07	0.02	
4	Intergenic	3	103166745	PZE-103062307	A,G	1.14E-06	0.021	0.09	0.03	
5	Intergenic	3	103544700	PZE-103062210	A,G	9.29E-06	0.039	0.17	0.02	
6	Intergenic	3	103769943	PZE-103062159	A,C	9.79E-06	0.039	0.13	0.02	
7	Intergenic	3	146026075	PZE-103087994	A,C	1.35E-05	0.040	0.3	0.02	
8	Intergenic	4	153495851	PZE-104079154	G,A	4.17E-06	0.039	0.25	0.03	
9	GRMZM2G080842	4	153499805	SYN13972	A,G	8.29E-06	0.039	0.24	0.02	Mitochondrial carrier protein
10	GRMZM2G080842	4	153500453	SYN13976	C,A	1.09E-05	0.039	0.24	0.02	Mitochondrial carrier protein
11	GRMZM2G080842	4	153500492	SYN13977	A,G	1.23E-05	0.040	0.23	0.02	Mitochondrial carrier protein
12	GRMZM2G080842	4	153501980	PZE-104079162	C,A	1.04E-05	0.039	0.25	0.02	Mitochondrial carrier protein
13	GRMZM2G080842	4	153502008	PZE-104079163	A,G	1.07E-05	0.039	0.24	0.02	Mitochondrial carrier protein
14	GRMZM2G171605	4	186590896	PZE-104110312	A,G	1.84E-06	0.021	0.18	0.03	4'phosphopantetheinyl transferase
15	GRMZM2G168807	5	165320067	SYN16674	A,C	1.50E-05	0.040	0.32	0.02	WW/Rsp5/WWP
16	Intergenic	5	187471551	PZE-105130754	A,G	1.47E-05	0.040	0.28	0.03	
17	GRMZM2G151651	7	33447828	SYNGENTA5726	G,A	1.79E-06	0.021	0.08	0.03	
18	GRMZM2G020254	10	65416520	PZE-110034333	A,G	1.24E-06	0.021	0.21	0.03	DNA-binding WRKY
19	GRMZM2G089484	10	88686456	PZE-110047506	G,A	1.59E-05	0.040	0.4	0.02	Tyrosine protein kinase

*False discovery rate-corrected p-values

^a^Minor allele frequency

### Haplotype-based association studies

**Fig. 3:**
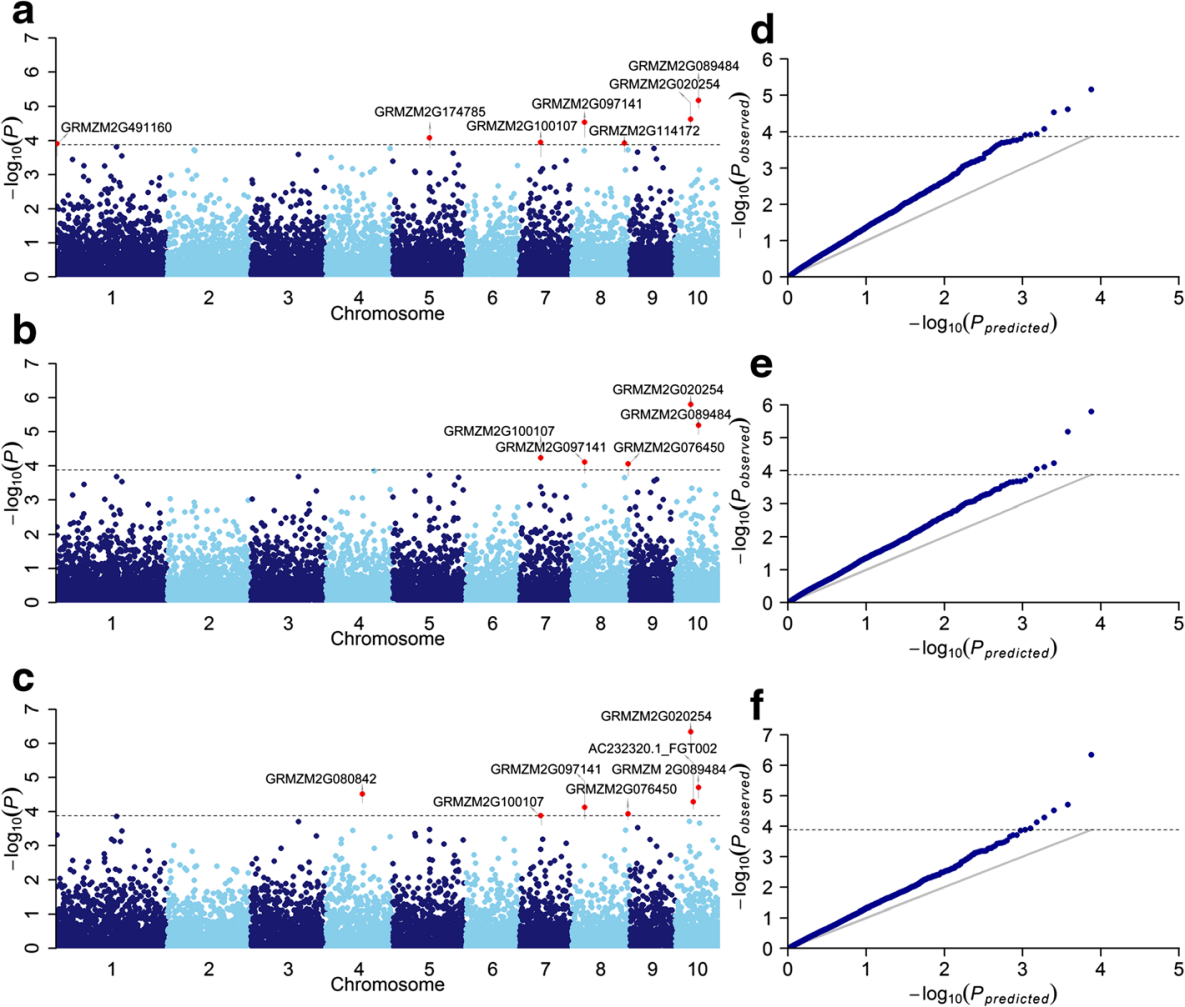
Manhattan plots and QQ plots resulting from the haplotype-based GWAS for AUDPC, Mean Rating and High Rating. Manhattan plots for area under disease progress curve (AUDPC), Mean Rating and High Rating are shown in **a**, **b** and **c**, respectively. QQ plots for area under disease progress curve (AUDPC), Mean Rating and High Rating are shown in **d**, **e** and **f**, respectively

Gene-based haplotypes were constructed within the 7,551 genes which had at least 2 SNPs. On average a set of 4.9 haplotypes was defined in each of the 7,551 genes in present study. The haplotype analysis using these loci and phenotypic data from three disease parameters (i.e., AUDPC, mean rating and high rating) identified ten loci associated with resistance to NCLB. Of these loci, seven, five and seven were significantly associated with AUDPC, mean rating and high rating (−log_10_*P* > 3.88, *P* = 1/7,551 loci), respectively (Fig. 3). Among the significant loci, four possible candidate genes (GRMZM2G089484, GRMZM2G020254, GRMZM2G097141 and GRMZM2G100107) were significantly associated with all three disease parameters (Table 5), and three of them were annotated as resistance-related proteins (tyrosine protein kinase, DNA-binding WRKY and SANT domain-associated). When comparing the loci identified by single-SNP and haplotype-based associations, identical loci were also detected. For example, two candidate genes (GRMZM2G100107 and GRMZM2G020254) were significantly associated with at least two disease parameters based on both haplotype-based and SNP-based association analyses.

**Table 5:** Chromosome, gene name and annotation of the genes for high rating, mean rating and AUDPC detected by haplotype-based GWAS

No.	Chromosome	Gene name	Traits	Annotation
1	1	GRMZM2G491160	AUDPC	
2	4	GRMZM2G080842	High Rating	Mitochondrial carrier protein
3	5	GRMZM2G174785	AUDPC	ENTH/VHS
4	7	GRMZM2G100107	High Rating, Mean Rating, AUDPC	SANT associated
5	8	GRMZM2G097141	High Rating, Mean Rating, AUDPC	
6	8	GRMZM2G114172	AUDPC	Ubiquitin
7	8	GRMZM2G076450	High Rating, Mean Rating	BTB/POZ-like
8	10	GRMZM2G020254	High Rating, Mean Rating, AUDPC	DNA-binding WRKY
9	10	AC232320.1_FGT002	High Rating	
10	10	GRMZM2G089484	High Rating, Mean Rating, AUDPC	Tyrosine protein kinase

### Anderson-Darling (A-D) test for genome scanning

The SNP data were further used for genome-wide scanning via A-D test to reveal the sources of resistance to NCLB. The total population was divided into three subgroups as described in the Methods section. Trait-marker association was performed by A-D test for each subgroup. As shown in the QQ and Manhattan plots (Additional file 5: Figure S2; Additional file 6: Figure S3; Additional file 7: Figure S4; Additional file 8: Figure S5), we found notable positive associations in subgroup 1, in which >100 significant markers associated with different disease parameters were observed. In contrast, few significant associations were revealed in subgroup 2 and only small number of significant associations was observed in subgroup 3. The predicted genes located within associated SNPs were identified using the MaizeGDB genome browser [16] or the http://ensembl.gramene.org/Zea_mays/Info/Index browser [17]. Here we listed 81 genes which were associated with at least two or three of the disease parameters (Additional file 9: Table S4). Among the predicted genes, 12 were related to plant defense (Table 6), which included antifreeze protein, PR transcriptional factor and a receptor-like kinase similar to those involved in basal defenses, and could be evaluated as potential candidate resistance genes. More importantly, when compared the defense genes with those identified by other two methods in present study (single-marker and haplotype-based associations), we found GRMZM2G100107 was identical for all three analyses, and GRMZM2G171605 was identical for A-D test and single-marker based associations.

**Table 6:** A subset of 81 SNP loci found to be associated with resistance to NCLB by Anderson-Darling test

No.	Chromosome	Physical position (AGP v.2)	Gene ID	Subpopulation	Traits	Predicted gene function
1	1	198469464	GRMZM2G123094	subpop-3	AUDPC, Mean Rating	Antifreeze protein
2	1	202300043	GRMZM2G315375	subpop-1	AUDPC, Mean Rating	ABC transporter
3	1	202549145	GRMZM2G112377	subpop-1	AUDPC, Mean Rating	Antifreeze protein
4	2	149335132	GRMZM2G124524	subpop-1	AUDPC, High Rating, Mean Rating	PR transcriptional factor
5	3	135911049	GRMZM2G153087	subpop-1	AUDPC, High Rating, Mean Rating	FYVE/PHD
6	3	145476628	GRMZM2G397948	subpop-1	AUDPC, High Rating, Mean Rating	BTB/POZ
7	4	40358905	GRMZM2G059266	subpop-1	AUDPC, High Rating, Mean Rating	Protein kinase C
8	4	186590896	GRMZM2G171605	subpop-1	AUDPC, High Rating, Mean Rating	4'phosphopantetheinyl transferase
9	7	7462912	GRMZM2G406859	subpop-1	AUDPC, High Rating, Mean Rating	Antifreeze protein
10	7	91684720	GRMZM2G100107	subpop-1	AUDPC, High Rating, Mean Rating	SANT associated
11	10	10290662	GRMZM2G093895	subpop-1	AUDPC, High Rating, Mean Rating	Transcription factor
12	10	116680462	GRMZM2G175525	subpop-3	AUDPC, High Rating, Mean Rating	PR transcriptional factor

## Discussion

Resistance to NCLB is a complex trait, and we know comparatively little about the genetic architecture in maize [18]. In the present study, a large number of lines were used to dissect the genetic architecture of resistance to NCLB. The germplasm covered a considerable amount of the genetic diversity found globally in maize, including 999 inbred lines from different sources, which were, most importantly, from multiple locations, allowing us to depict a clear global image. The high heritabilities of traits associated with resistance to NCLB revealed the potential of this panel for precisely mapping NCLB resistance genes. However, the population structure of the association panel is an important factor for GWAS. To minimize spurious correlations and associations attributable to genetic non-independence or genome-wide linkage disequilibrium (LD), we unified significant population structure information (contained in matrix Q) and pairwise relative kinship relationships among lines (contained in matrix K) into the statistical model [19]. These results can significantly control the false positives, but the Q + K model was extremely strict, and it was hard to find significant loci when using the Bonferroni threshold as the cutoff (data not shown). Therefore, we used a PCA + K instead of Q + K model and observed significant loci for this disease. We further confirmed our results through different analysis methods, including a haplotype-based GWAS and A-D tests for genome scanning. We observed several genes using different statistical approaches and determined that some of the genes were commonly associated with all of the traits based on highly correlated phenotypic data. Furthermore, the genes detected in our investigation caused minor effects and controlled a small portion of phenotypic variation. Therefore, we concluded that resistance to NCLB is controlled by several genes or QTLs, each of which has a minor effect, and that no single major gene that controls NCLB resistance is present in this germplasm.

Several qualitative genes have been identified in tropical and temperate germplasm backgrounds that confer resistance to NCLB. Most of these *Ht* genes (for *Helminthosporium turcicum*, the former name of *E. turcicum*) are dominant or partially dominant, including *Ht1*, *Ht2*, *Ht3*, *Ht4*, *Htn1*, *Htm1* [20] and the more recently identified *HtP*, as well as *rt* [21]. Most of the genes were not cloned but mapped on chromosomes: *Ht1* and *HtP* were mapped on the long arm of chromosome 2 (bin 2.08) [22, 23], *Ht2* and *Htn1* were mapped on the bins 8.05 and 8.06 [24, 25] and *rt* was mapped on chromosome 3L (bin 3.06) [23]. We compared the physical locations of the predicted genes in the present study with the mapped *Ht* genes, and we found that *HtP* was closely linked with GRMZM2G139463 and *rt* was closely linked with GRMZM2G072780. More studies were required to understand the associations between the identified candidates and underlying genes. No doubt, present data provides good information for final cloning and validating these genes. Recently, two major QTLs, one on chromosome 1 (*qNLB1.06*_*Tx303*_) [26, 27] and the other on chromosome 8 (*qNLB8.06*_*DK888*_), which is closely linked and functionally related to *Ht2* [28], have been fine-mapped and their locations narrowed to 3.6 Mb and 0.46 Mb, respectively. However, we did not identify predicted genes within these regions in our population. Since high heritability of resistance to NCLB was observed in the association panel comprising of large number of lines, the major reason may be the number of markers in the population was limited(~50k). It was estimated that several million markers are required for a whole genome wide association study in maize [29], which makes us have no enough power to detect all the underlying loci affecting target traits.

Compared with single-marker association, haplotype-based association is expected to improve the power of detection when the marker density is limited. In the present study, the efficiency of LD mapping was improved by using a haplotype-based analysis, which was constructed from multiple SNP markers within the same gene. As a result, we identified a total of ten loci at a genome-wide level for the three disease parameters. Haplotypes may have the potential to be in higher LD with the causative variants than individual SNPs, especially when using medium-density SNP panels. Indeed, compared with the high heritabilities of the three traits, it was unlikely that resistance to NCLB was determined by only a small number of genes. It is more likely that resistance to NCLB is a complex trait involving a large number of loci, of which the candidates identified in this study may have the largest effects. Given the expected >50,000 maize genes and the 5–10 feasible SNPs per gene for a given haplotype, more markers are needed for precise LD mapping to accelerate the discovery of NCLB resistance genes in maize.

As we mentioned earlier, association mapping is a powerful tool to detect loci involved in the inheritance of traits, but identifying loci responsible for more complex traits is difficult. Population structure can result in spurious associations that result from unlinked markers being associated with causative loci [30]. Such associations can occur when the disease frequency varies across subpopulations, thus increasing the probability that affected individuals will be sampled. Any marker alleles that are present at a high frequency in the overrepresented subpopulation will be associated with the phenotype [31]. Recently, the A-D test was applied as a useful complement to GWAS of complex quantitative traits [32]. In present study, large number of markers was identified as having strong associations with the phenotype in the largest subgroup (subgroup 1), whereas the other two subgroups with less lines revealed few or small number of significant SNPs. Predicted genes containing the significant SNPs were identified, and 81 genes, including 12 genes that related to plant defenses, were found to be associated with two or three of the disease parameters. The A-D test balances false positives and statistical power, and it can be used to analyze complex traits such as resistance to NCLB in maize.

## Conclusion

An association panel including 999 diverse lines was evaluated for resistance to NCLB in multiple environments, and a large number of resistant lines were identified and can be used as reliable resistance resource in maize breeding program. GWAS reveals that NCLB resistance is a complex trait under the control of many minor genes with relatively small effects. Identical genes for resistance to NCLB were detected using single-marker and haplotype-based associations, as well as A-D test. Pyramiding these genes in the same background may result in stable resistance to NCLB.

## Methods

### Germplasm and phenotyping

The population used in this study represents the global collection of maize germplasm consisting of 999 inbred lines of a diverse nature. Three types of inbred lines, CMLs, CIMBLs (CIMMYT breeding lines) and the Drought Tolerant Maize for Africa (DTMA) lines, from the CIMMYT germplasm collection were used in this study (Additional file 1: Table S1). These lines were evaluated at 12 locations during two consecutive years under artificially created epiphytotics of *Exserohilum turcicum* (Additional file 2: Table S2). A randomized complete block design was used at all locations with a maximum of three replications per location. Each plot consisted of a single 2-m row with 10 plants. Inocula for field inoculations were produced with sterile sorghum grains. Briefly, a population of a pure *Exserohilum turcicum* strain was obtained from infected leaves collected from the preceding year following the procedure of Asea et al. [33]. Pure cultures were grown on PDA medium and used to inoculate sterile sorghum grains to produce large volumes of inoculum. Inoculated bottles containing sterile sorghum were cultured at room temperature for 2 weeks, and then colonized grains were harvested and kept in the dark at room temperature until use.

Experimental plots were inoculated at the 4- to 6-leaf stage by placing 20–30 grains of *Exserohilum turcicum*-colonized sorghum in the leaf whorl. Data on disease severity were recorded, as were the corresponding diseased leaf areas of each plant. Whole plots were visually rated three times during the growing season for the percent NCLB severity using the CIMMYT scale (1–5), where 1.0 = complete resistance, no lesions; 1.5 = very slight infection, one to a few scattered lesions on lower leaves, covering 0–5 % of the leaf surface only; 2.0 = weak-to-moderate infection on lower leaves with a few scattered lesions on lower leaves, covering 6–20 %; 3.0 = moderate infection, abundant lesions on lower leaves and a few on middle leaves, with 21–50 % of the leaf surface showing NCLB symptoms; 4.0 = abundant lesions on lower and middle leaves extending to upper leaves, covering 51–80 % of the leaf surface and 5.0 = abundant lesions on all leaves, plant may be prematurely killed, lesions covering >80 % of the leaf surface [34].

### Statistical analyses

The phenotypic multi-environmental data were subjected to the following methods to analyze different parameters. To minimize the effect of environmental variation, best linear unbiased prediction (BLUP) of each line were used for all three traits. BLUP estimation was by the model: y = Xb + Zu + e, where X and Z are incidence matrices. In general, b represents fixed effects, u represents random effects and e represents residuals. It is assumed that expectation are E(y) = Xb, E(u) = 0, E(e) = 0. Residuals are independently distributed with variance, so V(e) = R, V(u) = G and COV(u, e) = 0. R and G are known positive definite matrices. Hence

$$ V\left[\begin{array}{c}\hfill u\hfill \\ {}\hfill e\hfill \end{array}\right]=\left[\begin{array}{cc}\hfill G\hfill & \hfill 0\hfill \\ {}\hfill 0\hfill & \hfill R\hfill \end{array}\right] $$

$$ {u}_i=\frac{\sigma_A^2}{\sigma_e^2+{\sigma}_A^2}\left({Y}_i-\mu \right) $$

*σ*_*A*_^2^ is variance of additive effects, *σ*_*e*_^2^ is variance of random effects, *Y*_*i*_ is phenotypic observation of the *i* individual and *μ* is overall mean. *u*_*i*_ is BLUP value [35]. Analysis of variance was performed using SAS (Release 9.1.3; SAS Institute, Cary, NC, USA). The heritability of distinct traits was calculated as the ratio of the total genotypic to total phenotypic variances [36]. The average scoring data were used to calculate the mean rating, and the individual average data of each score at 7-day intervals was converted to the percent leaf area for the computation of AUDPC based on the formula suggested by Ceballos et al. [37] using the midpoint rule. AUDPC = Σ_i = 1_^n–1^ [(t_i + 1_–t_i_) (y_i_ + y_i+1_)/2], where t is the time in days of each reading, y is the percentage of affected foliage at each reading and n is the number of readings.

### Genotyping

Genomic DNA extraction was performed using a modified CTAB protocol [38]. At least five leaves from each line were pooled and used for DNA extraction. All 999 lines were genotyped using GoldenGate assays (Illumina, San Diego, CA, USA) that were comprised of 56,110 authenticated SNPs, which were derived from the B73 reference sequence, evenly distributed across the 10 maize chromosomes [39]. The SNP genotyping was performed on an Illumina Infinium SNP genotyping platform at Cornell University Life Sciences Core Laboratories Center using the protocol developed by the Illumina Company.

### Population structure

Population structure was estimated using the Bayesian Markov Chain Monte Carlo (MCMC) implemented in STRUCTURE [40, 41]. Briefly, SNPs with minor allelic frequencies ≥ 0.3 were used first to select major SNPs, and then 1,000 markers were randomly selected from the whole set based on the physical length of each chromosome. Hypotheses were tested for subpopulations number from *K* = 1 to *K* = 10. For each *K* value, seven independent runs were performed under the admixture model and correlated allele frequencies, with burn in time and MCMC replication number both to 100,000. The *K* value was determined by LnP(D) and hoc statistic delta *K* based on the rate of change of LnP(D) between successive *K* value [42]. Based on the simulation summary, bar plots were constructed with the lower value of var[LnP(D)], and the populations were divided into three subgroups based on the delta *K* following Yang et al. [43]. PCA was generated by setting the Genome Association and Prediction Integrated Tool-R package [44] and the K matrix was calculated using SPAGeDi software [45].

### SNP-based genome-wide association mapping

To use the best quality data for different analyses, we did not analyze data from several lines that had high levels of missing genotypic data. In total, 981 lines were used in the final analysis, and all of the lines had high-quality phenotypic and genotypic data. SNP-based genome-wide association mapping was determined by using TASSEL (Trait Analysis by Association, Evolution and Linkage) software [46]. Of the 56,110 SNPs genotyped, 46,451 SNPs with minor allelic frequencies ≥ 5 % were used for the GWAS. The MLM (PCA + K) model, which incorporated a kinship matrix (K) along with the covariate PC (the first 10 principal components), was performed using MLM (P3D, no compression) [19, 43]. P value of each SNP was calculated and significance was defined at a uniform threshold of P ≤ 2.15 × 10^−5^ (*P* = 1/n; *n* = total markers used, which is roughly a Bonferroni correction). SNP with the lowest P value was reported for each significant locus, and the predicted genes located within associated SNPs were identified using the MaizeGDB genome browser [16] or the www.maizesequence.org/genome browser [17].

### Haplotype-based association studies

In this study, SNP genotypes within the genes were selected to construct gene-based haplotypes. Since the number of SNPs in each gene varied (i.e., from one to fifteen), the genes which had only one SNP were discarded, and thus 7551 genes, each had ≥2 SNPs, were selected to construct the haplotypes. Briefly, the genome was divided into gene-based windows to determine the haplotypes of the linked SNPs. Each gene-based window was defined by all of the SNPs within a specific gene. If the gene contained more than five SNPs, a random subset of five SNPs was selected for the window. For subsequent analyses, each haplotype window was defined as a locus. Thus, 7551 gene-based windows were defined. Since there are more than one haplotypes within each gene, haplotypes with frequencies <5 % were discarded, then a multi-allelic test was performed for each set of haplotypes at a locus to identify the association between genes and traits. Haplotype-based GWAS was performed by using TASSEL software, and MLM was selected by taking both population structure PC (the first 10 principal components) and kinship (K) into account to avoid spurious associations.

### Anderson darling test

Anderson-Darling test is a nonparametric statistical method and a variation of the Kolmogorov-Smirnov test [47] that gives weight to the tails of the distribution. In present study, Anderson-Darling test was conducted in each of three subgroups of the association panel. Briefly, each subpopulation was subjected to the k-sample A-D (k = number of samples) test, which is a variation of the Kolmogorov-Smirnov test [47] for genome screening. The observed P value was used to construct QQ and Manhattan plots with SAS. The full details of this test have been published recently to dissect the genetic architecture of maize for 17 traits [32], and the software of A-D test can be performed using an R script and downloaded from http://www.maizego.org.

## Additional files

Additional file 1: Table S1. The list of the lines and their phenotypic evaluation to NCLB in the association panel. (ODS 58 kb) Additional file 2: Table S2. The field design of the association mapping panel for evaluation of resistance to NCLB. (ODS 13 kb) Additional file 3: Table S3. The proportion of variance explained by ten groups of principal component analyses in association panel. (ODS 12 kb) Additional file 4: Figure S1. Analysis of the population structure of maize inbred lines. **a**) Estimated LnP(D) and Δ k of STRUCTURE analysis; **b**, **c** and **d** show the genetic variance controlled by the 56110 SNP makers for AUDPC, Mean Rating and High Rating, respectively. (DOC 119 kb) Additional file 5: Figure S2. Manhattan plot for AUDPC in sub-group 1, 2 and 3, based on Anderson-Darling test. (DOC 66 kb) Additional file 6: Figure S3. Manhattan plot for Mean Rating in sub-group 1, 2 and 3, based on Anderson-Darling test. (DOC 64 kb) Additional file 7: Figure S4. Manhattan plot for High Rating in sub-group 1, 2 and 3, based on Anderson-Darling test. (DOC 66 kb) Additional file 8: Figure S5. QQ plot for all the traits using Anderson-Darling test. The QQ plot for sub-groups 1, 2 and, 3 were shown in blue, green and red colors, respectively; while black line is the expected line. (DOC 42 kb) Additional file 9: Table S4. SNP loci found to be associated with resistance to NCLB by GWAS using Anderson-Darling test. For the three disease parameters (AUDPC, mean rating and high rating), significant SNPs associated to 2 or 3 disease parameters were listed. (ODS 22 kb)

## Abbreviations

A-D test: Anderson-Darling test
AUDPC: Area under disease progress curve
CIMBL: CIMMYT maize breeding line
CML: CIMMYT maize line
DTMA: Drought Tolerant Maize for Africa
GWAS: Genome wide association studies
K: Kinship
LD: Linkage disequilibrium
MLM: Mixed linear model
NCLB: Northern corn leaf blight
PCA: Principal component analyses
Q: Structure
QQ: Quantile-quantile
QTL: Quantitative trait locus
SNP: Single-nucleotide polymorphism
