# Supplementary figures and images for: Genome-wide association mapping reveals novel sources of resistance to northern corn leaf blight in maize
Source: BMC Plant Biol. 2015 Aug 20;15:206. doi: 10.1186/s12870-015-0589-z (PMC4546088; doi:10.1186/s12870-015-0589-z)

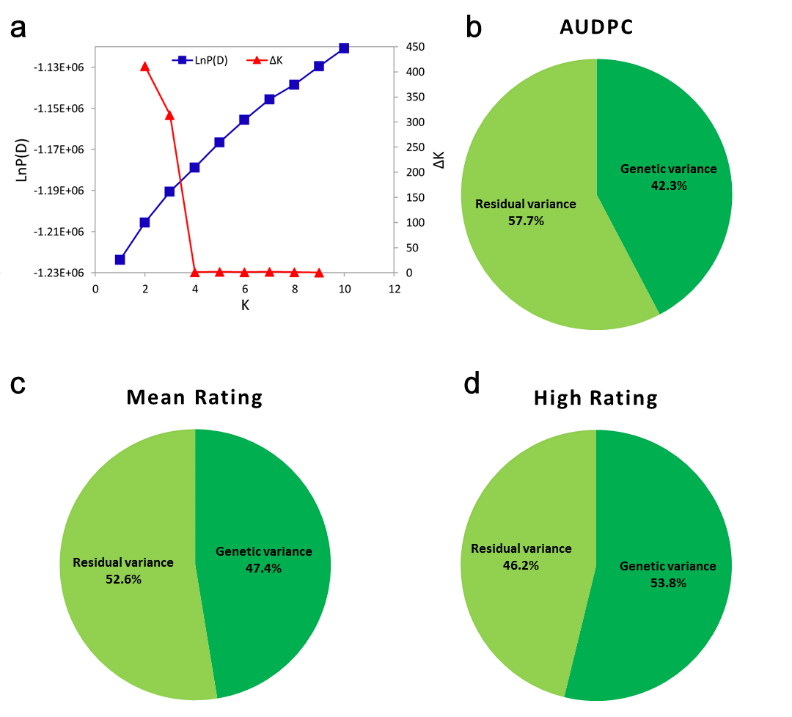


**Additional file 4: Figure S1.** Structure and control of genetic variance by the SNP markers.

Supplement: Additional file 4: Figure S1. — Analysis of the population structure of maize inbred lines. a) Estimated LnP(D) and Δ k of STRUCTURE analysis; b, c and d show the genetic variance controlled by the 56110 SNP makers for AUDPC, Mean Rating and High Rating, respectively. (DOC 119 kb) [file 12870_2015_589_MOESM4_ESM.doc]

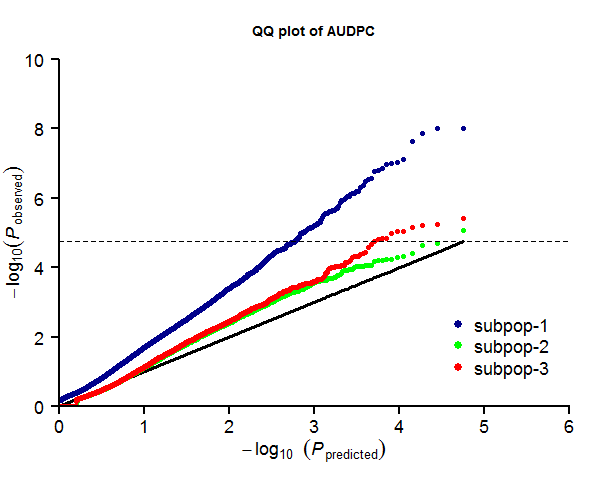


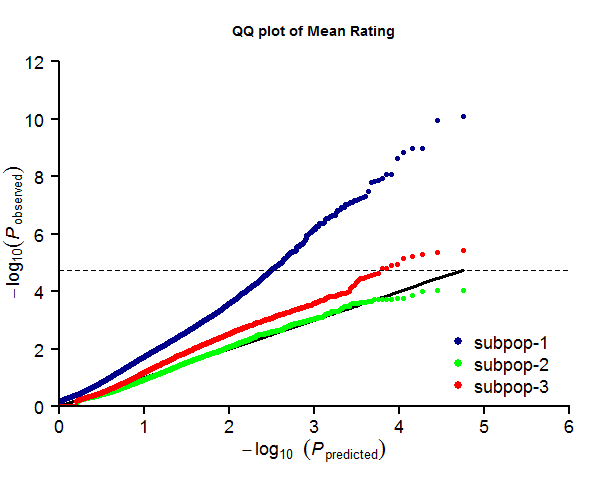


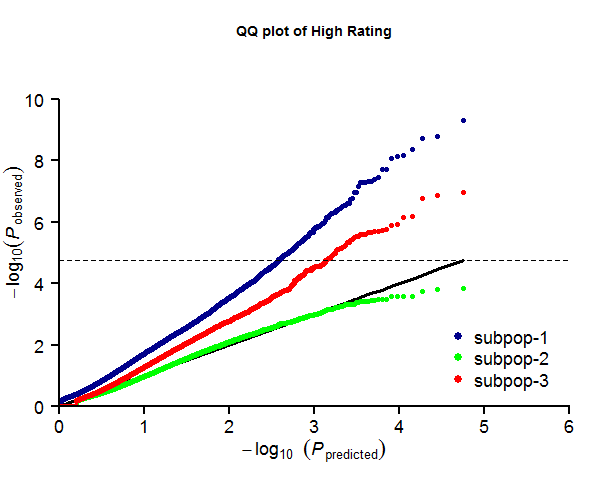


**Additional file 8: Figure S5.** QQ plot for all the traits using A-D test.

Supplement: Additional file 8: Figure S5. — QQ plot for all the traits using Anderson-Darling test. The QQ plot for sub-groups 1, 2 and, 3 were shown in blue, green and red colors, respectively; while black line is the expected line. (DOC 42 kb) [file 12870_2015_589_MOESM8_ESM.doc]
