# Supplementary material for: Genome-wide association mapping reveals novel sources of resistance to northern corn leaf blight in maize
Source: BMC Plant Biol. 2015 Aug 20;15:206. doi: 10.1186/s12870-015-0589-z (PMC4546088; doi:10.1186/s12870-015-0589-z)
Supplement: Additional file 5: Figure S2. — Manhattan plot for AUDPC in sub-group 1, 2 and 3, based on Anderson-Darling test. (DOC 66 kb) [file 12870_2015_589_MOESM5_ESM.doc]

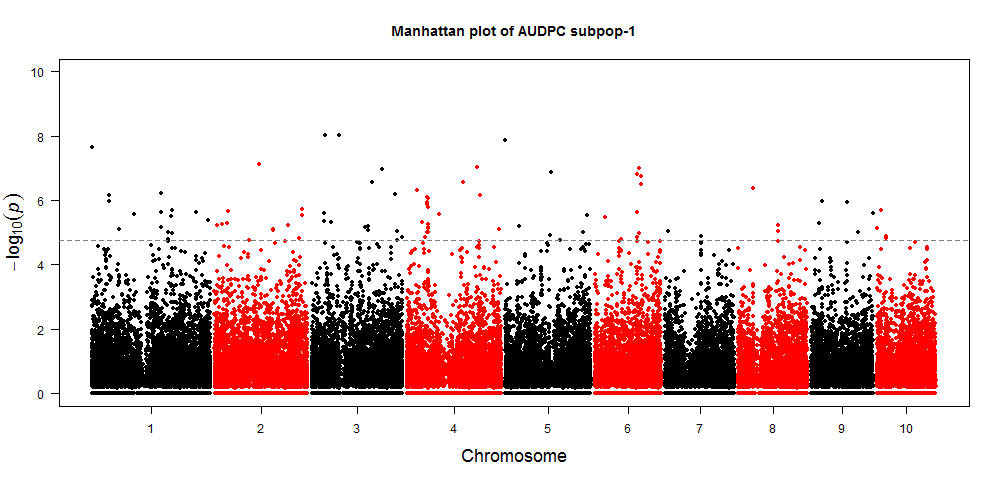

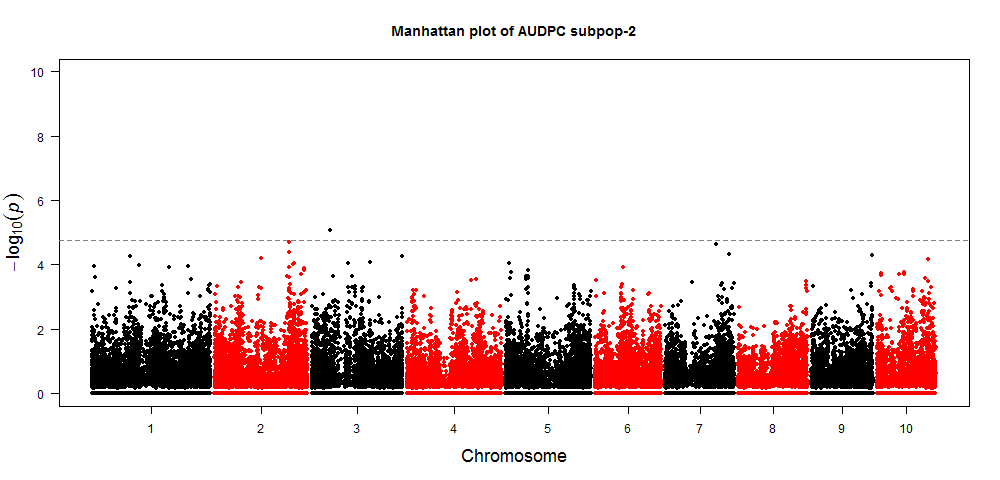


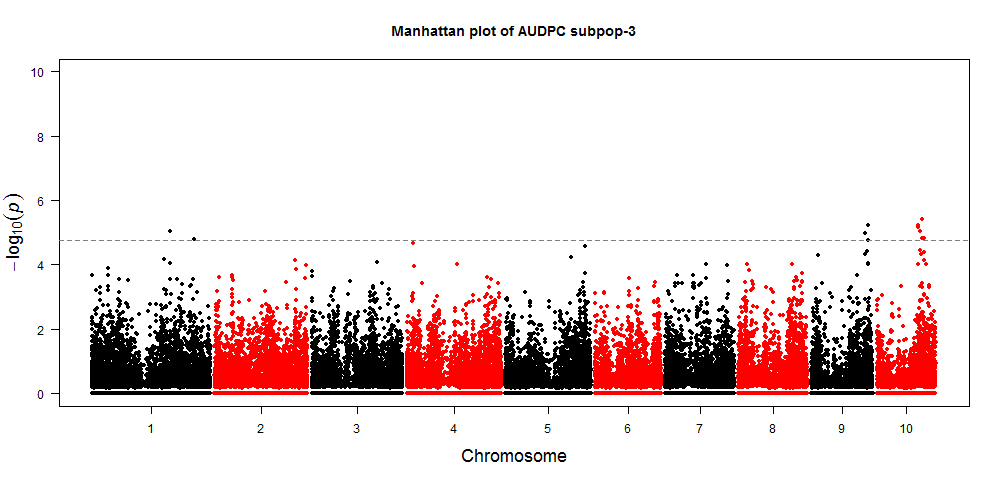


**Additional file 5: Figure S2.** Manhattan plot for AUDPC in sub-group 1, 2 and 3, based on Anderson-Darling test.
